# Aquaporins during Pregnancy: Their Function and Significance

**DOI:** 10.3390/ijms18122593

**Published:** 2017-12-01

**Authors:** Eszter Ducza, Adrienn Csányi, Róbert Gáspár

**Affiliations:** Department of Pharmacodynamics and Biopharmacy, Faculty of Pharmacy, University of Szeged, H-6720 Szeged, Hungary; ducza@pharm.u-szeged.hu (E.D.); csanyi.adrienn@pharm.u-szeged.hu (A.C.)

**Keywords:** aquaporin, water channel, pregnancy

## Abstract

Water is the major component of cells and tissues, and the movement of water across the cell membrane is a fundamental property of life. Until the discovery of the first water channel, aquaporin, it was long assumed that the transport of water was due to simple diffusion through the lipid bilayer membrane that encloses cells. Aquaporin (AQP) molecules were first discovered in the human uterus in 1994, and since then several studies have investigated these channels in the female reproductive system. The expressions of AQPs have been proven in the reproductive system. Their levels are altered during the implantation process, both in the uterus and the fetal cells, and participate in the control of the flow of amniotic fluid. They seem to be very important for the normal placental functions. AQPs are present during parturition, participating in the control of pregnant myometrial contractions and cervical ripening. However, most of the physiological and regulatory roles of AQPs are not clarified in the reproductive tract. Furthermore, no satisfactory knowledge is available about their sensitivities to different drugs. AQP-selective ligands may contribute to the development of new drug candidates and the therapy of several reproductive disorders.

## 1. Introduction

Aquaporin (AQP) water channels are responsible for enabling the rapid passive movement of water across a membrane. These small hydrophobic integral membrane proteins are divided into three groups. Classical AQPs, including AQP0, 1, 2, 4, 5, 6, and 8, are selectively permeable to water [1]. Aquaglyceroporins are AQP3, 7, 9, and 10, which allow the transport of water and non-polar solutes (e.g., urea or glycerol), reactive oxygen species (e.g., hydrogen peroxide), gases (e.g., ammonia, carbon dioxide, nitric oxide), and metalloids [2,3,4]. Unorthodox aquaporins include AQP11 and 12; their functions are not clearly identified [5,6]. Many of them have been identified in mammalian reproductive systems [7].

The structural data have revealed that the functional AQP unit is a homotetramer [8], and that each AQP monomer is composed of six transmembrane α-helices connected by alternating intracellular and extracellular loops. The transmembrane domains form a right-handed bundle around the central pore of each AQP monomer, through which water transport occurs. The specificity of the pore for water is a result of direct hydrogen binding between single-file water molecules and the AQP family’s signature Asn-Pro-Ala motif at the narrowest part of the pore. Water selectivity is further aided by interactions with the aromatic/arginine constriction site, which physically restricts the pore [9].

The normal water homeostasis in the female reproductive system is indispensable for healthy pregnancy and successful birth. AQPs could play a remarkable role in this homeostasis during the whole gestational period.

## 2. Implantation

The regulation of luminal fluid is important for blastocyst implantation. The reduction in the amount of luminal fluid at the time of implantation contributes to the close opposition between the trophoblastic and luminal epithelial cells, and is essential for successful implantation [10]. An increase in AQP1 expression was found in mesometrial myometrium at the time of implantation, where it may contribute to the implantation position of the blastocyst in rats and mice. It is supposed that AQP1 expression in the myometrium may play a role in the closure of the uterine lumen as well as the orientation of the blastocyst at the time of implantation [11,12,13]. It is well known that progesterone has a crucial role in implantation, and this sex hormone up-regulates the AQP1 expression in the inner circular layer of myometrium in ovariectomized rats [12]. In humans, AQP2 was found in glandular and luminal epithelial cells, with an increased expression during the mid-secretory phase, which corresponds to the time of implantation [14]. In a microarray study, AQP3 was found in human uterine luminal epithelial cells; however, AQP2 was not detectable in these human uterine samples [15]. The endometrial expression of AQP4 is upregulated by the presence of a day 7 embryo in the cranial portion of the uterine horn of cattle, indicating the reprogramming of water transport before hatching [16]. Sun et al. proved that the female AQP4 knockout mice had reduced fertility and weak ovulatory response to human choriogonadotropin (hCG)-induced superovulation treatment. In a lack of AQP4, even the uterine weight and thickness were significantly lower after 14 h of hCG injection [17]. More studies demonstrated the presence of AQP5 in the mouse and rat uterus, where AQP5 was present in the apical plasma membrane of luminal epithelial cells and there is an increase in expression in mesometrial epithelial cells at the time of implantation [18,19]. Furthermore, the appearance of AQP5 in the apical plasma membrane was dependent on progesterone [12].

By in situ hybridization in mice, AQP8 mRNA was localized in the inner cell mass, while AQP9 mRNA was found in the mural trophectoderm of the blastocyst. AQP8 mRNA was also detected in decidualised stroma and receding uterine glands in the mouse uterus [20]. AQP8 deficiency increases the fertility of female mice because it increases the number of mature follicles by reducing the apoptosis of granulosa cells [21].

AQP1–7, 9, 11, and 12 are expressed in human luteinized granulose cells from infertile women undergoing a stimulated in vitro fertilization cycle. The mRNA level of AQP1 was negatively associated with retrieved oocyte number, while the mRNA level of AQP7 was positively associated with fertilization rate [22].

The expression of AQP9 protein in the epithelial cells of the fallopian tube was significantly reduced during pathological types of implantation such as human tubal pregnancy revealed by immunohistochemistry. This reduced expression of AQP9 was not related to estrogen receptor or progesterone receptor expression [23]. In contrast, the estrogen and estrogen agonist treatment dramatically increased AQP5 and AQP8 mRNA expressions in the mice uterus and the intrauterine fluid volume, and as a result, the sites of implantation decreased. Progesterone (P4) supplementation could neutralize the adverse effect of the elevated estrogen level. It can be important in respect of in vitro fertilization because during this procedure the standard ovarian hyperstimulation could cause supraphysiological levels of steroid hormones such as estrogen (E2), leading to altered E2/P4 ratio and impaired and aberrant implantation [24].

The polyphenolic compound quercetin has an estrogen-like effect. It is present in high amounts in fruits and vegetables. Quercetin stimulates fluid accumulation in the uterus and down-regulates the expression of AQP5 and AQP9 in the uterus; thereby, it could adversely affect embryo implantation in rat [25]. This finding is contradictory as compared with other estrogen compounds. However, it must be emphasized, that quercetin has direct, estrogen-independent action on the ion transport and fluid secretion that might be responsible for the phenomenon [26].

A. Skowronska et al. proved that arachidonic acid, forskolin, and cAMP up-regulated AQP1 and AQP5 expression in the porcine uterus during the implantation period (days 14–16 of pregnancy) in uterine explants studies. AQP5 expression was down-regulated by oxytocin. Estrogen (3 h) treatment resulted in a rapid decrease in AQP1 and 5 expressions, while long (24 h) treatment elevated AQP5 and 1 gene expression in the porcine uterus. This makes the significance of long-term estrogen exposition more obvious in up-regulation of AQP1 and AQP5 as compared with short estrogen exposition. After long exposition, the changes of AQP5 were in the basolateral plasma membrane of the epithelial cells, suggesting transcellular water movement between the uterine lumen and blood vessels [27].

Based on the above-mentioned facts, the fluid reabsorption at the time of implantation is essential for the increase in implantation rate with a reduction in luminal fluid. However, the presence of different AQP isoforms seem species-specific in these processes.

## 3. Maternal–Fetal Fluid Flow

### 3.1. Amniotic Membrane

The amniotic membrane plays several roles in the regulation of amniotic fluid flow during pregnancy. This transport process through the intramembranous route has two components: a unidirectional vesicular transcytotic transport of amniotic water with dissolved solutes out across the amnion cell; and a passive diffusion of water down osmotic gradients from amniotic fluid into fetal blood, presumably through aquaporin channels [28]. AQPs have additional roles in cell proliferation, migration, urea transport, and apoptosis of amniotic membrane cells. AQP1, 3, 8, 9 were determined and investigated in mouse, ovine, and human amniotic membranes. In mice, AQP1 was localized in the plasma membrane of amniotic fibroblasts, AQP3 was in both epithelial and fibroblasts cells, AQP8 was expressed in epithelial cells, and AQP9 was only in the apoptotic cells of the epithelium [29]. It was proved that of the five determined AQPs in the amniotic membrane, AQP1 has a positive role in regulating the rate of fluid transfer across the ovine amnion. AQP11 protein is expressed in large quantities in human amnion and chorionic cells at the end of pregnancy (38 weeks of pregnancy) [30]. AQP11 is considered as an unorthodox aquaporin because its first asparagine–proline–alanine (NPA) motif has an unusual sequence—the alanine is substituted by cysteine. However, this difference does not negatively affect the water permeability of AQP11 as compared with AQP1 [5]. AQP11 has been found in the testis, liver, kidney, muscle, heart, and brain. The biological functions of AQP11 are thought to be related to the endoplasmic reticulum [31]. It was ascertained that AQP11-knockout mice develop polycystic kidney disease and die prematurely, suggesting the importance of AQP11 in intravesicular homeostasis [32].

Bouvier et al. investigated AQPs in fetal membranes from diabetic pregnant women, because polyhydramnios is a relatively common complication in pregnant women with diabetes. They ascertained that the expression of AQP3 was significantly lower in groups of mothers with type 2 diabetes and gestational diabetes [33].

### 3.2. Placenta

The amniotic fluid volume is regulated across the placenta, and this maternal–fetal interaction is essential during pregnancy. The volume abnormalities (polyhydramnios or oligohydramnios) are associated with increased fetal morbidity and mortality. AQP1, 3, and 8 expressions are increased in the second half of pregnancy in ovine placentas. AQP1 is localized in the vasculature, while AQP3 and 8 are found in the trophoblast epithelial cells of the placenta. AQP1 is the major gene expressed at the beginning of pregnancy, when the trophoblast formation is minimal; in contrast, the expression of AQP3 and 8 is at its maximum at the end of pregnancy in sheep [34]. AQP4, the most abundant water channel in the brain, was also determined in human placenta. Its expression decreases in the syncytiotrophoblast from the first to the third trimester of gestation, and increases in endothelial cells and the stroma of placental villi [35].

Escobar et al. investigated the chorionic villi samples between the 10th and 14th weeks of human gestation. They found high mRNA expression for AQP1, 3, 9, and 11, low for AQP4, 5, and 8, and AQP2, 6, and 7 were undetectable in chorionic villi. High expression of AQP11 was identified in early stages of human pregnancy. Chromosomal abnormalities did not influence the expression of AQPs in human chorionic villi [36].

Oligo- and polyhydramnios have been considered a potential sign of fetal mortality, and are associated with a higher frequency of adverse prenatal outcome. AQP8 and 9 were examined in fetal membrane and placenta in term human pregnancy complicated by polyhydramnios and oligohydramnios. In polyhydramnios cases, the expression of AQP8 in the amnion and that of AQP9 in the amnion and the chorion were significantly increased in the idiopathic polyhydramnios group; however, their expression in the placenta was significantly decreased [37]. In human term fetal membranes and placenta with oligohydramnios, the expression of both AQP8 and AQP9 was significantly decreased in the amnion, while their expressions in the placenta were significantly increased. The AQP9 level was also significantly decreased in the chorion, while that of AQP8 was unchanged [38].

Maternal undernutrition also influences the AQP expression in rat placenta. AQP1 expression is significantly decreased, while AQP8 and 9 expressions are significantly increased. This kind of dysregulation may represent a compensatory mechanism for the occurrence of oligohydramnios during early maternal undernutrition [39].

The AQP9 expression level is increased in the placenta during gestational diabetes, which is regulated in a dose-dependent manner by leptin in human trophoblast explants studies. AQP9 could mediate the increased transport of glycerol to the fetus, providing for the increased energy intake requirements in the macrosomic fetus [40].

AQP2 expression is higher in the placenta of patients with severe preeclampsia than in normal pregnant women. Moreover, the positive correlation of the AQP2 level with human chorionic gonadotropin was found in severe preeclampsia [41]. Another study proved that AQP3 has a critical role in the regulation of placental apoptosis in pathological placentas (e.g., in preeclampsia). The loss of water via AQP3 from the cells during the apoptotic volume decrease is important to the progression of apoptosis [42].

## 4. Parturition

### 4.1. Myometrial Contraction

Our group investigated the presence and pharmacological reactivity of AQP isotypes in the late pregnant rat uterus. We determined AQP1, 2, 3, 5, 8, and 9 from day 18 to day 22 of pregnancy. During pregnancy, the expression of AQP5 was much higher as compared with other AQPs, however its expression was dramatically decreased on the last day of gestation. It was also proved that this type of water channel is selectively up-regulated by oxytocin [43]. Our results were confirmed by microarray analysis, in which the significant down-regulation of AQP5 mRNA was determined by about 45- to 100-fold during delivery, underscoring their potential role in parturition in the rat uterus. Microarray analysis during pregnancy revealed that AQP5 and 8 were up-regulated during the first 20 days of pregnancy [44]. We demonstrated that AQP5 expression is influenced by both estrogen and progesterone in the late-pregnant rat uterus. Progesterone and progesterone derivatives up-regulated AQP5 expression, and it was more predominant than estrogen. This finding was confirmed by hormonally-induced (antigestagen with prostaglandin E2) preterm labour, resulting in a significant drop in the expression of AQP5 in the pregnant rat uterus. The decreasing effect of preterm delivery on AQP5 expression was similar to the last day of natural pregnancy. So, we suggest that reduced AQP5 expression after progesterone deprivation may contribute to the initiation of preterm labour [45].

### 4.2. Cervical Ripening

The increased cervical water content results in changes in cervical connective tissue and a shift in glycosaminoglycan composition. The process of cervical ripening is a specific phase of remodeling associated with a loss of structural integrity and tensile strength, which enables the cervix to dilate at the end of pregnancy. The presence of AQP3, 4, 5, and 8 was determined in mice pregnant cervix. The peak expression of AQP3 was on day 19 of pregnancy. AQP4 expression was low during pregnancy, and the AQP5 and 8 levels decreased at the end of pregnancy in mice. Lipopolysaccharide (LPS)-induced preterm labor had similar trends in AQP4, 5, and 8 expressions in mice with normal labor at term. AQP3 was preferentially expressed in basal cell layers of the cervical epithelium, whereas AQP4, 5, and 8 were primarily expressed in apical cell layers [46]. It was proved that relaxin increases hyaluronan synthase 2 and AQP3 in the cervix of late pregnant mice, so AQP3 may have a role in the water transport into the extracellular space, reducing collagen density and promoting cervical ripening [47].

## 5. Conclusions

Since their discoveries in the uterus, AQPs have been intensively investigated in the reproductive system. The expressions of AQPs have been identified by molecular biological and pharmacological methods in different species (Table 1).

However, several questions can be raised about the water channels, such as their physiological and regulatory roles in the reproductive tract. Furthermore, limited information is available about their pharmacological properties. Currently, pharmacological examinations of AQPs are almost impossible because of the lack of non-toxic tissue- and subtype-selective agonists or antagonists. So far, few compounds have been proposed to modulate both classical and aquaglyceroporin types of AQPs. AQP-selective ligands may promote the better clarification of the roles of AQPs in several reproductive disorders.

## Figures and Tables

**Table 1:** The expression of aquaporin (AQP) isotypes (P: protein or R: mRNA) in different mammalian species (h: human; r: rat; m: mouse; c: cattle; o: ovine; p: pig) during pregnancy. aec: apoptotic epithelial cells; acl: apical cell layers of cervical epithelium; bcl: basal cell layers of cervical epithelium; bz: basal zone; cp: complete placenta; ec: epithelial cells; em: endometrium; fc: fibroblast cells; gc: granulose cells; icm: inner-cell mass of blastocytes; lz: labyrinth zone, mm: myometrium; mt: mural trophectoderm of blastocyst; pv: placenta villi; st: syncytiotrophoblast cells; tec: trophoblast epithelial cells; vec: vascular epithelial cells.

Sites of Expression	AQP1	AQP2	AQP3	AQP4	AQP5	AQP6	AQP7	AQP8	AQP9	AQP11	AQP12
Oocytes/blastocytes (implantation)	h-gc-R	h-gc-R	h-gc-R	h-gc-R	h-gc-R	h-gc-R	h-gc-R	m-icm-P	h-gc-R m-mt-P	h-gc-R	h-gc-R
Uterus (implantation)	r-mm-P m-mm-P+R p-P	h-em-P+R	h-em-P+R	m-mm-P+R c-em-R	m-mm-P+R r-mm-P p-P	-	-	m-mm-P	r-P+R p-P	-	-
Amnionic membrane	h-P+R o-P+R m-fc-P	-	h-P+R o-P+R m-ec, fc-P	-	-	-	-	h-P+R o-P+R m-ec-P	h-P+R o-P+R m-aec-P	h-P+R o-P+R	-
Placenta	h-pv-R r-bz, lz-P m-vec-P+R o-vec-P+R	-	h-pv-R r-R m-tec-P+R o-tec-P+R	h-pv-R h-st, ec-P	h-pv-R	-	-	h-pv-R r-bz, lz-P m-P+R o-tec-R	h-pv-R r-cp, bz, lz-P m-R	h-pv-R	-
Uterus (late pregnant)	r-P+R	r-P+R	r-P+R		r-P+R	-	-	r-P+R	r-P+R	-	-
Cervix (late pregnant)	-	-	m-bcl-P+R	m-acl P+R	m-acl P+R	-	-	m-acl P+R	-	-	-
